# Targeting stem cell niche can protect hematopoietic stem cells from chemotherapy and G-CSF treatment

**DOI:** 10.1186/s13287-015-0164-4

**Published:** 2015-09-15

**Authors:** Sidan Li, Dehui Zou, Changhong Li, Hengxing Meng, Weiwei Sui, Sizhou Feng, Tao Cheng, Qiongli Zhai, Lugui Qiu

**Affiliations:** State Key Laboratory of Experimental Hematology, Institute of Hematology and Hospital of Blood Diseases, Chinese Academy of Medical Sciences and Peking Union of Medical College, 288 Nanjing Road, Tianjin, 30020 China; Beijing Key Laboratory of Pediatric Hematology Oncology, National Key Discipline of Pediatrics, Ministry of Education, Hematology Oncology Center, Beijing Children’s Hospital, Capital Medical University, Beijing, China; Department of Pathology, Tianjin Medical University Cancer Institute and Hospital, National Clinical Research Center for Cancer, Key Laboratory of Cancer Prevention and Therapy, Tianjin, 300060 China

## Abstract

**Introduction:**

Hematopoietic stem/progenitor cells (HSPCs) reside in a tightly controlled local microenvironment called bone marrow niche. The specialized microenvironment or niche not only provides a favorable habitat for HSPC maintenance and development but also governs stem cell function.

**Method:**

We investigated the effect of cytotoxic drugs on bone marrow niche. To mimic the multiple rounds of chemotherapy followed by autologous hematopoietic stem cells (HSCs) transplantation in a clinical setting, we further verified the hypothesis that targeting the niche might improve stem cell–based therapies in mouse models.

**Results:**

We found that multiple rounds of cytotoxic drug treatment significantly disrupted niche and serum osteocalcin level was significantly reduced after treatment in autologous HSPCs transplanted patients (*P* = 0.01). In mouse models, the number of CD45^−^Ter119^−^OPN^+^ osteoblasts was significantly reduced after multiple rounds of chemotherapies and granulocyte colony stimulating factor (G-CSF) treatment (P < 0.01). Parathyroid hormone (PTH) or receptor activator of nuclear factor kappa-B ligand (RANKL) treatment significantly increased the number of HSCs mobilized into peripheral blood (PB) for stem cell harvesting and protected stem cells from repeated exposure to cytotoxic chemotherapy. Treatments with G-CSF and PTH significantly increased the preservation of the HSC pool (P < 0.05). Moreover, recipient mice transplanted with circulation HSPCs that were previously treated with PTH and RANKL showed robust myeloid and lymphatic cell engraftment compared to the mice transplanted with HSCs after chemotherapy or G-CSF treatment.

**Conclusion:**

These data provide new evidence that the niche may be an important target for drug-based stem cell therapy.

## Introduction

Hematopoietic stem cell transplantation (SCT) has provided lifesaving treatment for many hematological disorders, but a significant proportion of patients who are eligible for autologous SCT fail to mobilize a sufficient number of CD34^+^ hematopoietic stem/progenitor cells (HSPCs), which is called “poor mobilization”, owing to various premobilization (predictive) factors such as prior treatment with stem cell toxic drugs, underlying disease, age, prior radiotherapy, and bone marrow involvement [1–3]. Poor mobilization has disastrous consequences for patients, with potential loss of transplant as a treatment option. Moreover, 5–10 % of healthy donors cannot obtain adequate HSPCs for allogenetic transplantation after granulocyte colony-stimulating factor (G-CSF) treatment [1, 4]. Repeated attempts during the mobilization process will increase resource use, but morbidity and patient/donor inconvenience are also increased in the meantime. How to improve the mobilization efficiency is therefore becoming a challenging topic for hematological scholars [5, 6].

Poor mobilization may result from significant depletion of the bone marrow hematopoietic stem cell (HSC) pool caused by G-CSF. Since the specialized microenvironment (niche) governs stem cell function [7, 8], targeting the stem cell niche may change the fate of stem cells. Our previous studies demonstrated that, except for the proteolytic enzymes, cellular components of osteoblasts and osteoclasts are closely related to G-CSF-induced HSPC mobilization in healthy donors [9, 10]. Recognition of the intimate relationship between endosteal niche cells (osteoblasts and osteoclasts) and HSPCs affords the possibility of targeting the niche to improve stem cell mobilization efficiency. The role of parathyroid hormone (PTH) in activating osteoblasts triggered researchers to investigate the possible effect of PTH on HSPCs. The pharmacological role of PTH in HPSCs during G-CSF-induced mobilization has been confirmed in a phase I clinical trial [11]. Moreover, it has been found that the resorption of osteoclasts stimulated by receptor activator of nuclear factor kappa-B ligand (RANKL) can reduce the level of stem cell niche components along the endosteum and finally trigger HSPC mobilization, so RANKL may be used together with other mobilization agents in clinical HSPC transplantation protocols [12]. In our previous study, PTH/RANKL was added to manipulatively interrupt the bone remodeling balance and then increase the number of HSPCs mobilized into the peripheral blood (PB). We demonstrated that the imbalance of bone remodeling can facilitate HSPC mobilization [9], and targeting the HSC niche may improve mobilization efficiency.

In this study, the role of bone remodeling in G-CSF-induced mobilization was examined in clinical specimens from autograft patients, and several animal models mimicking clinical mobilization situations were established to explore the possibility of improving poor mobilization.

## Materials and methods

### Sample collection

PB samples from 10 autograft patients (including three non-Hodgkin’s lymphoma (NHL) cases, two myeloma cases, and five cases with acute lymphoblastic leukemia (ALL)) were first collected after diagnosis (before treatment). A median of four chemotherapy cycles (range 3–6 cycles) was then administered to these patients [13–15]. Before the mobilization course, PB samples were again collected from patients (steady state). All patients were autografted in the first remission. The mobilization course consisted of subcutaneous injection of human recombinant G-CSF (5 μg/kg/day, twice a day, Filgrastim; Japan) used in the recovery phase of myelotoxic chemotherapy (single-agent cyclophosphamide (China) infusion or mitoxantrone (China) plus cytarabine (China)). Serum samples were collected by centrifugation at 500 × *g* for 10 minutes and stored at −80 °C for assay. Human samples were used in accordance with approved procedures by the Human Experimentation and Ethics Committee of the Institute of Hematology and Blood Diseases Hospital, Chinese Academy of Medical Sciences (CAMS) and Peking Union of Medical College (PUMC). We obtained consent from all patients involved in the study, including consent to participate in the study where appropriate.

### In vivo experiment

**Fig. 1:**
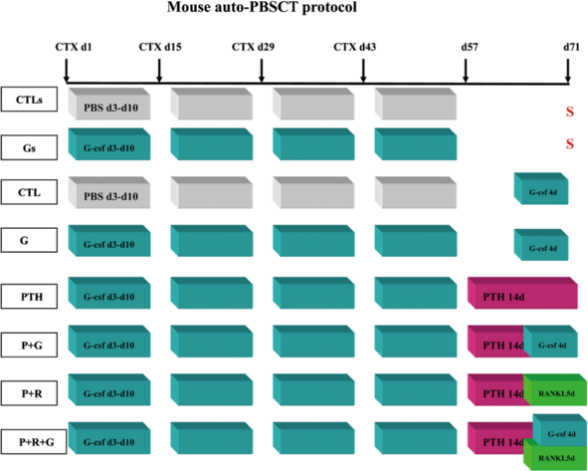
Diagrammatic representation of the experimental procedure to analyze the effects of PTH (80 μg/kg/day, Bachem, Torrance, CA) and RANKL (6 μg/day, PeproTech, Rocky Hill, NJ) treatment during multiple rounds of chemotherapy with cyclophosphamide (CTX,5 mg) and/or G-CSF, 250 μg/kg/day. In the last 2 weeks, one-half of the CTL and G groups received no treatment. At the end of the 10-week treatment period, mice were scarified (S), and bone marrow HSPCs (CTL / G / PTH) and HSPCs mobilized into the blood (CTL / G / P + G / P + R / P + R + G) were assessed by competitive repopulation assay (*n* = 6–8 each group)

C57Bl/6 and B6.SJL female mice (6–8 weeks old) were obtained from the Institute of Laboratory Animal Science, CAMS and PUMC. All of the animal handling and experimental procedures were approved by the Animal Care and Use Committee of CAMS and PUMC. Mice were housed in sterilized micro-isolator cages and received autoclaved food and water. To study the changes of niche cells and HSPCs after cytotoxic drug treatment, C57Bl/6 (CD 45.2) mice were injected intraperitoneally with cyclophosphamide (CTX) (Sigma, Sigma-Aldrich, St Louis, MO, USA) at a dose of 5 mg once every 2 weeks for a total of four cycles, and then injected intraperitoneally with saline (CTLs group) or recombinant human G-CSF (Filgrastim) (Gs group) at a dose of 250 μg/kg/day for 8 consecutive days. At the end of 10 weeks (day 71, 8-week treatment period and 2-week recovery period), mice were killed and the functions of osteoblast and HSPCs were tested (Fig. 1).

To imitate the settings of autologous transplantation following chemotherapy, after each of the four cyclophosphamide treatments already mentioned C57B1/6 mice were injected intraperitoneally with either saline (group CTL) or 250 μg/kg/day G-CSF (Filgrastim, groups G, PTH, P + G, P + R, and P + R + G) for 8 consecutive days. After 8-week treatments, mice were treated with G-CSF for 4 days (Filgrastim, 250 μg/kg/day, intraperitoneally, groups CTL and G), or rat PTH for 14 days (80 μg/kg/day, intraperitoneally, group PTH; Bachem, Torrance, CA), or a combination of PTH for 14 days and G-CSF for 4 days (group P + G), or a combination of PTH for 14 days and RANKL for 5 days (6 μg/day, subcutaneous injection via the femur, group P + R; PeproTech, Rocky Hill, NJ), or a combination of PTH for 14 days, RANKL for 5 days, and G-CSF for 4 days (group P + R + G). At the end of treatments, mice were sacrificed, and bone marrow HSPCs (groups CTL, G, and PTH) and HSPCs mobilized into the blood (groups CTL, G, P + G, P + R, and P + R + G) were assessed by competitive repopulation assay (CRA) (shown in Fig. 1).

### Isolation of bone marrow nuclear cells and osteoblasts

Total bone marrow nuclear cells were isolated from mouse femurs by flushing with phosphate-buffered saline (PBS) plus 2 % fetal bovine serum. Red blood cells and debris were removed by ammonium-chloride–potassium (ACK) lysis (0.15 M NH_4_Cl, 1.0 mM KHCO_3_, 0.1 mM ethylenediamine tetraacetic acid (EDTA), pH 7.4) and filtering through nylon mesh. Osteoblasts were isolated from marrow-depleted femurs by mechanical disruption (crushing with mortar and pestle), infused with PBS containing 50 mg/ml type II collagenase (GIBCO, NY) and incubated at 37 °C for 15 minutes. The collagenase-treated femurs were flushed with PBS and a similar procedure was repeated six times. Osteoblasts were collected by centrifugation at 400 × *g* for 5 minutes and cells were pooled [16].

### Measurements of osteocalcin and tartrate-resistant acid phosphatase 5b levels

Serum levels of osteocalcin and tartrate-resistant acid phosphatase 5b (TRACP 5b) from mouse model and healthy donors samples were tested using immunoassay kits for osteocalcin (Biomedical Technologies, Stoughton, MA, USA for mouse; Immunodiagnostic Systems Limited, Boldon, UK for human) and TRACP 5b (Immunodiagnostic Systems Limited for mouse) following the manufacturer’s instructions. The concentrations of osteocalcin and TRACP 5b in each sample were calculated based on the average of different dilutions and the experiments were repeated three times.

### Competitive repopulation assay

For qualitative measurement of the HSC frequency in the peripheral circulation, 300 μl PB were collected from the retro-orbital vein of the C57Bl/6 (CD 45.2) mouse. PB was collected into microtainer tubes containing lithium heparin. The red cells were lysed with ACK lysing buffer and the mononuclear cells were mixed with 2.5 × 10^5^ bone marrow mononuclear cells from two B6.SJL (CD 45.1) mice. To measure the bone marrow HSC cell frequency, the mice were killed with carbon dioxide and the bone marrow mononuclear cells were isolated by flushing the bone marrow cavity with PBS plus 2 % fetal bovine serum. Then 2.5 × 10^5^ bone marrow mononuclear cells from C57Bl/6 mice were mixed with an equal number of bone marrow mononuclear cells from two B6.SJL competitor mice. These two kinds of hybrid cells were then injected into recipient B6.SJL mice that were lethally irradiated for 24 hours with 9.5 Gy radiation. The relative contribution of engraftment from the different cell sources was assessed by the detection of CD45.2 antigens in both the myeloid (defined as Side Scatter^hi^Mac-1^+^) and lymphoid (defined as Side Scatter^lo^CD3^+^/B220^+^) fraction of cells after 16 weeks. The cells were diluted and incubated with phycoerythrin (PE)-conjugated CD45.2, fluorescein isothiocyanate (FITC)-conjugated CD3, allophycocyanin (APC)-conjugated B220, and PE-Cy5-conjugated Mac-1 antibodies (eBiosciences, San Diego, CA, USA). After incubation with these antibodies, the samples were fixed and red cells were removed using BD fluorescence-activated cell sorting (FACS) lysis solution (BD Biosciences, San Jose, CA, USA).

### Flow cytometry

To quantify osteoblast lineage cells, bone-associated cells obtained from enzymatic treatment were stained with APC-conjugated anti-mouse CD45 and Ter119 antibodies (eBiosciences, San Diego, CA, USA), and goat anti-mouse osteopontin, followed by FITC-conjugate donkey anti-goat IgG (Santa Cruz Biotechnology, Inc., Texas, USA). CD45^−^/Ter119^−^/OPN^+^ cells were enumerated [17]. The population of different cells was assessed by FACS Calibur flow cytometer and analyzed with Cell Quest software (Becton-Dickinson).

### Colony formation assay

Nucleated bone marrow cells (1.0 × 10^4^) were planted in 2.5 ml methylcellulose media supplemented with a cocktail of recombinant cytokines (MethoCult 3434; StemCell Technologies, Vancouver, BC, Canada). Cultures were plated in duplicate and cultured in a humidified chamber with 5 % carbon dioxide at 37 °C. Colonies with at least 50 cells were counted on day 12 of culture.

### Reverse transcription and real-time quantitative PCR

Mouse marrow-depleted bones were flushed with a total of 1 ml TRIzol reagent (Invitrogen, Carlsbad, CA, USA) followed by crushing of the remaining bone in TRIzol. Reverse transcription was carried out using the Superscript First-Strand Synthesis System (Invitrogen) following the manufacturer’s instructions. Real-time quantitative PCR for osteocalcin (forward primer, 5′-TCTCTCTGCTCACTCTGCTGGCC-3′; reverse primer, 5′-TTTGTCAGACTCAGGGCCGC-3′) expression was performed on the ABI 7500 Sequence Detection System (Applied Biosystems, Foster, CA, USA). The 20 μl PCR mixture consisted of 10 μl Power SYBR® Green PCR Master mix (Applied Biosystems), 0.5 μl each primer (100 μM), 1 μl cDNA (40 ng RNA), and 8 μl ddH_2_O. The reaction was carried out 95 °C for 15 minutes, followed by 40 cycles of 95 °C for 15 seconds and 60 °C for 1 minute. ΔΔCT values were calculated from the differences between the targeted genes and internal control β-actin. Each experiment was repeated three times and the mean was calculated.

### Immunohistochemistry

To mark osteoblasts, immunohistochemical staining of osteocalcin was performed on formalin-fixed, paraffin-embedded sections of human biopsy specimen samples. Immunohistochemistry was carried out following the standard protocols. After dewaxing and antigen retrieval, the sections were blocked with goat serum for 1 hour and incubated with mouse anti-osteocalcin monoclonal antibody (1:100, ab13418; Abcam, Cambridge, UK) overnight at 4 °C. The sections were stained with streptavidin-peroxidase method and a 3,3′-diaminobenzidine (DAB, Venata Medical Systems, Basal, Switzerland) kit.

The osteoblast enumeration was performed in the growth region of all trabecular bones, but not any cortical bone (using 20× objective). Two pathologists counted the positive cells in 15 fields (400×) per section in a blind fashion. The number of osteoblasts was averaged and signified as the osteoblast number per bone surface.

### Hematoxylin and eosin and TRACP staining

Mouse femurs were fixed in 4 % formaldehyde in PBS for 48 hours, decalcified in 10 % EDTA (pH 7.5) for 14 days, and embedded in paraffin. Sections (4 μm) were deparaffinized, rehydrated, and stained with hematoxylin and eosin (H&E) and TRACP immunocytochemistry kit (Sigma-Aldrich Ltd, Dorset, UK) according to the manufacturer’s instructions for histochemical examination [18,19].

### Statistical analysis

The statistical significance of overall differences among multiple groups was analyzed by the ordinary analysis of variance using SPSS 15.0 (IBM, Chicago, IL, USA). Data are presented as the mean ± standard error of the mean. Data were analyzed using the nonparametric Mann–Whitney test as appropriate for the data set. *P* <0.05 was considered statistically significant.

## Results

### Multiple treatments of cytotoxic drugs destroy bone marrow niche osteoblasts in autologous transplantation patients

**Fig. 2:**
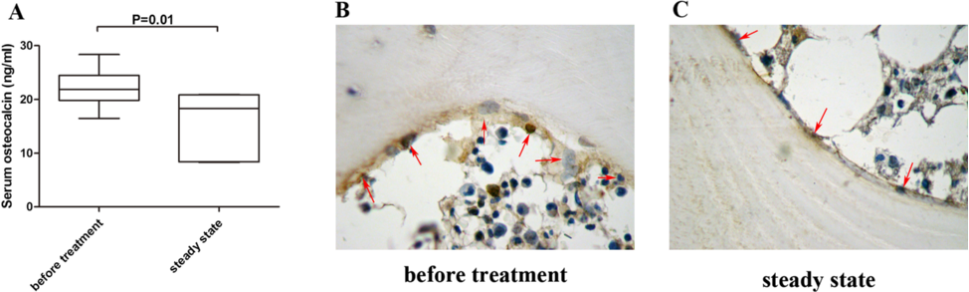
Chemotherapy destroys bone marrow niche osteoblasts in autologous transplantation patients. **a** Serum osteocalcin concentrations decrease after multiple rounds of cytotoxic drug treatment in human. Serum samples were collected from 10 patients and their serum osteocalcin levels were measured by enzyme-linked immunosorbent assay (ELISA). **b, c** Representative photomicrographs of human endosteal osteoblasts after immunohistochemical staining. Osteocalcin-positive mature osteoblasts (*red arrows*) before treatment (**b**) were considerably reduced at steady state (**c**) of G-CSF treatment

Based on our previous results, a mobilization protocol with G-CSF suppresses osteoblast function [9]. In this study, we further investigated the effect of multiple treatments of cytotoxic drugs on niche cells. PB samples from 10 patients were collected after diagnosis (before treatment) and before the mobilization chemotherapy course (steady state). We found that the osteocalcin level in the serum was significantly reduced after treatment (22.19 ± 1.08 ng/ml before treatment vs. 16.08 ± 2.12 ng/ml steady state, *P* = 0.01) (Fig. 2a). Moreover, the number of mature osteoblasts was significantly decreased after multiple chemotherapy cycles, which is defined by osteocalcin-positive endosteum cells (Fig. 2b, c). The number of osteoblasts per bone surface was decreased from 18.55 ± 0.32 (before treatment) to 12.27 ± 0.66 (steady state) (*P* <0.05). These data indicate that cytotoxic drugs not only decrease the number of osteoblasts, but also suppress the activity of osteoblasts.

### Multiple treatments of cytotoxic drugs influence both osteoblasts and HSPCs in a mouse model

**Fig. 3:**
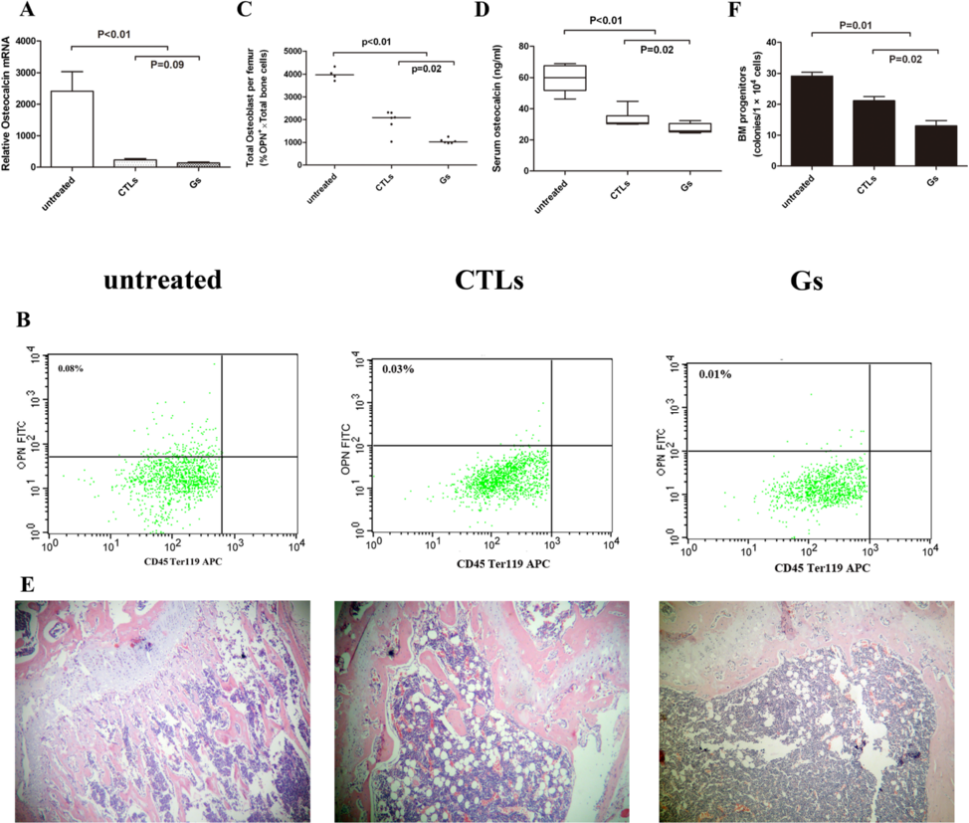
Multiple treatments with cytotoxic drugs influence both osteoblasts and HSPCs in a mouse model. **a** Osteocalcin mRNA levels were significantly decreased after four cycles of cyclophosphamide treatment, with or without supportive G-CSF therapy (*n* = 5–8 each group). **b** Representative scatter plots showing the CD45^−^Ter119^−^OPN^+^ osteoblast population isolated from C57Bl/6 mice. **c** Number of CD45^−^Ter119^−^OPN^+^ cells from the femurs of C57Bl/6 mice after four cycles of cyclophosphamide treatment, with or without supportive G-CSF therapy (*n* = 4–6 each group). **d** Serum osteocalcin concentrations decreased after four cycles of cyclophosphamide treatment, with or without supportive G-CSF. The osteocalcin levels were measured by ELISA (n = 6–8 each group). **e** H&E staining of sections of decalcified proximal femur from the untreated, CTLs and Gs groups (original magnification, ×40). **f** The number of HSPCs (colony-forming cells) in the bone marrow of C57Bl/6 mice reduced significantly (*n* = 6 each group)

To verify the results from patients, two mouse models (CTLs and Gs groups) were established by cyclophosphamide treatments and consecutive stimulations with G-CSF to mimic the patients who received high-dose chemotherapy after autologous PB SCT. Compared with untreated mice, osteocalcin mRNA expression was reduced 9.32 ± 0.3-fold in the CTLs group (*P* <0.01) and 16.82 ± 0.8-fold in thex Gs group (*P* <0.01) (Fig. 3a). The number of CD45^−^Ter119^−^OPN^+^ osteoblasts (%CD45^−^Ter119^−^OPN^+^ × total bone cells per femur) was significantly reduced after cyclophosphamide treatments and consecutive stimulations with G-CSF (untreated, 3993 ± 129 cells/femur; CTLs, 1937 ± 196 cells/femur; Gs, 1055 ± 43 cells/femur; *P* <0.01) (Fig. 3b, c). Moreover, the osteoblastic activity decreased to a low level. Circulation osteocalcin was decreased in the CTLs group (33.81 ± 1.99 ng/ml) and the Gs group (27.18 ± 1.09 ng/ml) compared with untreated mice (59.44 ± 3.16 ng/ml) (*P* < 0.01; Fig. 3d). In addition, the decreased number of trabeculae was observed in the long bones from the mice in both the CTLs and Gs groups (Fig. 3e). Furthermore, compared with untreated mice (29.17 ± 1.22 U), the number of HSPCs in the bone marrow was significantly reduced in the CTL (21.16 ± 1.35 U) and Gs (13.00 ± 1.71 U) groups (*P* = 0.01) (Fig. 3f). These results indicate that chemotherapy, especially in combination with G-CSF, can destroy the osteoblastic niche and lead to a significant depletion of the bone marrow HSPC pool, which may be the primary cause for poor mobilization.

### PTH and RANKL can efficiently activate the functions of osteoblasts and osteoclasts during cytotoxic chemotherapy

**Fig. 4:**
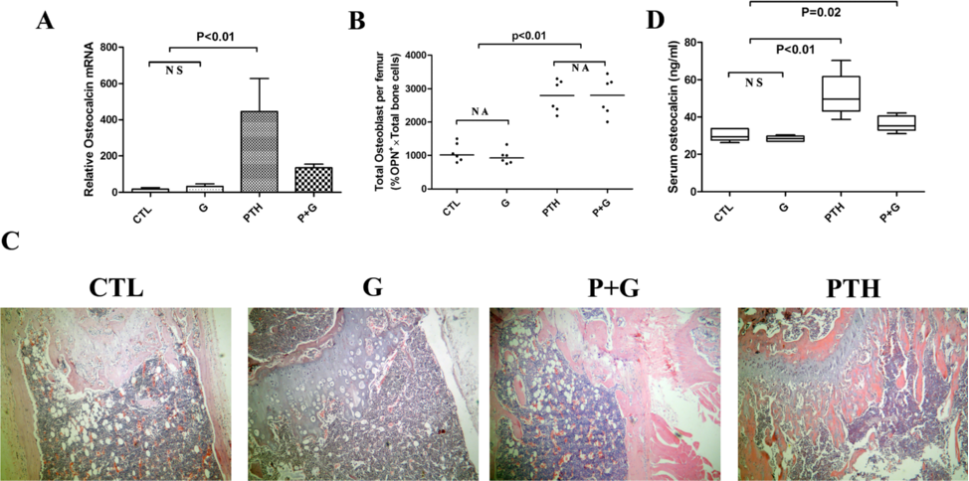
PTH can efficiently activate the functions of osteoblasts during cytotoxic chemotherapy. **a** Osteocalcin mRNA level were higher in PTH and P + G group compared with CTL or G group (*n* = 6–8 each group). **b** Number of CD45^−^Ter119^−^OPN^+^ cells from the femurs of C57Bl/6 mice (*n* = 6 each group). **c** H&E staining of sections of decalcified proximal femur from untreated, CTL, G, PTH and P + G groups (original magnification, ×40). **d** Serum osteocalcin concentrations of CTL, G, PTH and P + G groups. The osteocalcin levels were measured by ELISA (*n* = 5 each group)

In this study, we found niche cells (osteoblasts and osteoclasts) involved in stem cell mobilization and cytotoxic drugs combined with G-CSF suppressed the function of osteoblasts and HSPCs, so we hypothesized that stimulation of the HSPC niche, rather than the stem cell itself, may provide therapeutic benefit for clinical SCT. Here we found that PTH-treated mice showed a significant increase in the absolute number and function of osteoblasts. The level of osteocalcin mRNA in the PTH and P + G groups was significantly higher than that in the CTL or G group (*P* <0.01; Fig. 4a). Consistent with gene expression analysis, the number of CD45^−^Ter119^−^OPN^+^ osteoblasts in the PTH (2780 ± 197 cells/femur) and P + G (2768 ± 236 cells/femur) groups was significantly higher than that in the CTL (1091 ± 114 cells/femur) and G (954 ± 87 cells/femur) groups (*P* <0.01; Fig. 4b). Moreover, the density of trabeculae in the long bones was significantly increased in the PTH and P + G groups (Fig. 4c). PTH treatment also enhanced osteoblast function. Of the two PTH-treated groups, the osteocalcin level in the PTH and P + G groups was 51.89 ± 5.17 ng/ml and 36.43 ± 1.89 ng/ml, respectively, while its level was 30.39 ± 1.47 ng/ml in the CTL group and 28.34 ± 0.65 ng/ml in the G group (*P* = 0.02; Fig. 4d).

**Fig. 5:**
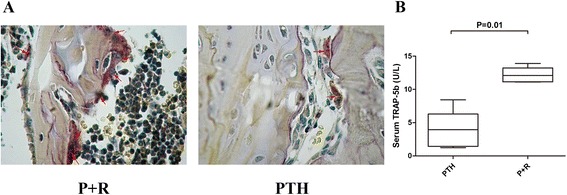
RANKL can efficiently activate the functions of osteoclasts during cytotoxic chemotherapy. **a** TRAP staining of mouse femoral metaphysis of PTH and P + R groups. *Arrowheads*: active TRAP^+^ osteoclasts stained in red (original magnification, ×400). **b** Serum TRAP-5b level also detected by ELISA (*n* = 5 each group). *P + R* mice injected with PTH and RANKL, *PTH* parathyroid hormone, TRAP 5b, tartrate-resistant acid phosphatase 5b

RANKL can stimulate osteoclasts and elevate the serum TRACP 5b level [15, 16]. Here we observed that RANKL treatment significantly increased the number of osteoclasts in the trabecula bone (P + R group vs*.* PTH group; Fig. 5a). The serum TRACP-5b level was also significantly elevated after RANKL treatment (12.16 ± 0.52 U/l) compared with PTH treatment only (3.87 ± 1.28 U/l) (*P* = 0.01; Fig. 5b). These data suggest that PTH may overcome the side effect of cytotoxic chemotherapy on osteoblasts. Moreover, RANKL treatment can efficiently activate osteoclast function.

### PTH and RANKL can protect and expand the resident HSC pool during cytotoxic chemotherapy

**Fig. 6:**
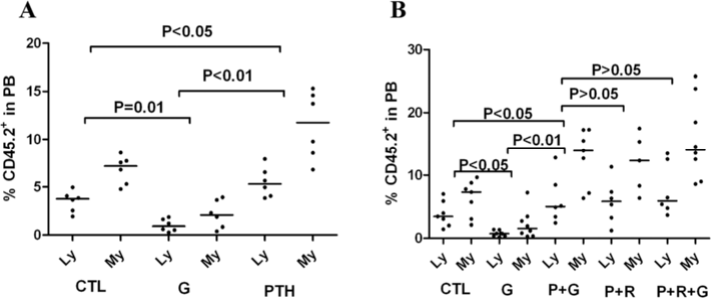
PTH and RANKL can protect and expand the resident HSC pool during cytotoxic chemotherapy. **a** Measurement of the HSC content of the bone marrow in the CTL, G and PTH groups by CRA at 16 weeks (*n* = 6 each group). **b** Measurement of the HSC content of the PB of mice in the CTL, G, P + G, P + R and P + R + G groups by CRA at 16 weeks (*n* = 6–8 each group)

Our previous data demonstrated that interrupting the balance of bone remodeling can facilitate HSPC mobilization [9]. In this study, the hematopoietic repopulation activity of HSCs and transplanted HSPCs was assessed at 16 weeks after transplantation by flow cytometric analysis of donor-derived (CD 45.2) myeloid, mature B and T cells in the recipients’ (CD 45.1) PB. CRA analysis of bone marrow HSPCs showed that there was a significant depletion of the HSC pool in the mice treated with G-CSF (CTL 11.2 % CD45.2^+^chimerism vs. G 4.8 % CD45.2^+^chimerism, *P =* 0.01) (Fig. 6a). However, treatment with PTH resulted in relative preservation of the HSC pool (G vs*.* PTH 16.7 % CD45.2^+^chimerism, *P* <0.01; CTL vs. PTH, *P* <0.05) (Fig. 6a). Similarly, analysis of the mobilization of HSCs into the peripheral circulation demonstrated that in mice who did not receive supportive G-CSF therapy during chemotherapy there was mobilization of HSCs into the circulation, while mice who received supportive G-CSF therapy alone showed little mobilization of HSCs into the peripheral circulation (CTL 10.8 % CD45.2^+^chimerism vs. G 3.3 % CD45.2^+^chimerism, *P* <0.05) (Fig. 6b). This was partially reversed by the following treatment with PTH (G vs*.* P + G 19.5 % CD45.2^+^chimerism, *P* <0.01; CTL vs. P + G, *P* <0.05) (Fig. 6b). Moreover, recipient mice transplanted with circulation HSPCs from the P + R and P + R + G groups showed more robust myeloid and lymphatic cell engraftment than those in the mice transplanted with HSCs from either the CTL or G group (Fig. 6b). RANKL could mobilize HSC efficiently as well as G-CSF (P + G vs*.* P + R 16.8 % CD45.2^+^chimerism, *P* >0.05) (Fig. 6b). However, RANKL treatment failed to amplify the mobilization of HSCs treated with PTH and G-CSF (P + G vs. P + R + G 17.5 % CD45.2^+^chimerism, *P* >0.05) (Fig. 6b). These data indicate that cytotoxic chemotherapy markedly depletes HSCs in bone marrow. Targeting the niche cells can protect and expand the resident HSC pool in the bone marrow during chemotherapy, and then effectively counteract the side effect of G-CSF.

## Discussion

In this study, the activity of bone marrow niche osteoblasts was detected in 10 autograft patients and several clinically relevant model systems. As expected, we found that compared with G-CSF treatment alone, cytotoxic chemotherapy combined with G-CSF treatment showed more severe inhibition of osteoblasts as well as HSPCs. Considering both PTH and RANKL are important regulators for bone remodeling [20–25], they were added to manipulatively interrupt the balance of bone remodeling. Finally, our data show that PTH and RANKL treatment can protect and even expand the resident HSC pool during cytotoxic chemotherapy.

HSCs reside within specific bone marrow niches and are anchored by adhesive interactions [26, 27], so repetitive cycles of chemotherapy may damage the niche and disrupt HSC functions such as quiescence, proliferation, and self-renewal [28]. During HSC mobilization in healthy donors, G-CSF significantly increases the number of HSCs in PB, but suppresses the number and the activities of osteoblasts in the meantime [5, 29, 30]. Our previous data have demonstrated the pivotal role of bone remodeling in the above processes, so it is reasonable to manipulate this balance to improve the mobilization efficiency [9]. During autologous SCT, a standard procedure has been applied, which involves multiple rounds of chemotherapy followed by G-CSF-induced mobilization of HSPCs [1]. How about bone remodeling in autologous SCT? Winkler et al*.* [28] demonstrated that the effects of cyclophosphamide and G-CSF in the metaphyseal spongiosa are similar to those of loss of osteoblasts, transient arrest in bone formation, and reduced CXCL12 expression. In this study, we also observed that the osteoblast niche was impaired by multiple rounds of cytotoxic drug treatment before G-CSF mobilization, in which the serum osteocalcin level obviously declined from 22.19 ± 1.08 ng/ml to 16.08 ± 2.12 ng/ml. We further verified these findings in two clinically relevant mouse models. The number of trabeculae and endosteal osteoblasts and the serum osteocalcin level in the long bones were significantly decreased in cyclophosphamide-treated mice, an effect that was further aggravated by G-CSF treatment. These data indicate that osteoblasts are destructed during autologous transplantation, even more severe than that in healthy donors. Moreover, multiple rounds of chemotherapy can significantly deplete the bone marrow HSPC pool, which is in accordance with the published report [5]. Protection of the niche function is therefore more important to ameliorate the poor mobilizers.

Considering the close proximity and importance of osteoblasts to HSPCs, we further demonstrated that both G-CSF and cyclophosphamide suppressed niche-supportive osteoblasts, and thus inhibited the expression of endosteal cytokines and resulted in major impairment of HSC reconstitution potential in the mobilized bone marrow. Based on the above findings, multiple rounds of cytotoxic chemotherapy (particularly when combined with G-CSF) impaired bone marrow function, and also limited the ability of patients to receive multiple rounds of optimal chemotherapy afterwards or limited the ability to obtain suitable stem cell products before a salvage bone marrow transplantation. Strategies to maintain the stem cell number and function in these clinical situations would therefore be desirable. Previous studies have demonstrated that both hematopoiesis and the HSC niche related to the bone remodeling process may be modulated by PTH and RANKL activity [31–33]. Our previous study also showed that, compared with G-CSF mobilization, an increase or decrease of the osteoblast/osteoclast ratio was closely related to the number of HSPCs in PB, suggesting that the imbalance of bone remodeling could facilitate HSPC mobilization [9]. To further verify whether the activation of endosteal niches can improve HSPC transplantation in a poor mobilization model, here we established six mouse models relevant to clinical uses of HSPCs. Our data showed that PTH treatment could increase the absolute number and function of osteoblasts, indicating that PTH may counteract the negative effects of cytotoxic drugs and G-CSF on osteoblasts. Moreover, we demonstrated that PTH treatment could increase the number of stem cells mobilized into PB and protect bone marrow stem cells from repeated exposure to cytotoxic chemotherapy and G-CSF. Furthermore, RANKL could also mobilize HSPCs to PB, so it may be used as a valuable alternative to G-CSF. Our data indicate that targeting the endosteal niche cells is a potential therapeutic approach to enhance stem cell-based therapies.

Our data are also supported by previous studies. Constitutive activation of the PTH receptor in osteoblasts increased the HSC number and activity [34]. Ballen et al*.* [35] performed a phase I study for PTH and found that 47 % of patients with hematologic malignancies acquired adequate CD34^+^ cells with the help of PTH. Moreover, RANKL-stimulated bone-resorbing osteoclasts reduce the stem cell niche components SDF-1, SCF, and OPN in the endosteum and finally trigger HSPC mobilization, so RANKL may be used together with other mobilization agents in an extensive range of clinical HSPC transplantation protocols [12]. In the present study, the CRA showed that recipient mice transplanted with circulation HSPCs from the P + R and P + R + G groups had more robust myeloid and lymphatic cell engraftment than either the CTL or G group. These findings suggest that stimulation of the endosteal bone marrow niche can lead to increased engraftment of the HSC compartment through increased expansion of the stem cell pool.

## Conclusions

Using clinical specimens and clinically relevant models, we demonstrate that manipulation of bone remodeling can increase the efficiency of HSC mobilization and targeting on the HSC niche is a viable and reasonable therapeutic choice in stem cell therapy. More importantly, our findings provide new knowledge for the development and treatment of poor mobilization.

## Abbreviations

ACK: Ammonium-chloride–potassium
ALL: Acute lymphoblastic leukemia
APC: Allophycocyanin
CAMS: Chinese Academy of Medical Sciences
CRA: Competitive repopulation assay
DAB: 3,3′-Diaminobenzidine
EDTA: Ethylenediamine tetraacetic acid
ELISA: Enzyme-linked immunosorbent assay
FACS: Fluorescence-activated cell sorting
FITC: Fluorescein isothiocyanate
G-CSF: Granulocyte colony-stimulating factor
H&E: Hematoxylin and eosin
HSC: Hematopoietic stem cell
HSPC: Hematopoietic stem/progenitor cell
NHL: Non-Hodgkin’s lymphoma
PB: Peripheral blood
PBS: Phosphate-buffered saline
PE: Phycoerythrin
PTH: Parathyroid hormone
PUMC: Peking Union of Medical College
RANKL: Receptor activator of nuclear factor kappa-B ligand
SCT: Stem cell transplantation
TRACP 5b: Tartrate-resistant acid phosphatase 5b
